# Comparison of surgical exoscope versus operating microscope in spine surgery: A Surgeon's perspective

**DOI:** 10.1016/j.bas.2025.104906

**Published:** 2025-09-06

**Authors:** Charlize Du Preez, Jake M. McDonnell, Aubrie M. Sowa, Keith Synnott, Marcus Timlin, Seamus Morris, Stacey Darwish, Joseph S. Butler

**Affiliations:** a UCD School of Medicine, University College Dublin, Belfield, Dublin, Ireland; b National Spinal Injuries Unit, Department of Trauma and Orthopaedic Surgery, Mater Misericordiae University Hospital, Dublin, Ireland

**Keywords:** Exoscope, Operating microscope, Spine surgery, Qualitative

## Abstract

**Introduction:**

A newly developed surgical visualization tool, the exoscope, has emerged as an alternative to the widely used operating microscope (OM). Through a qualitative survey, this study aimed to assess surgeons' perspectives on the exoscope compared to the OM, identifying its advantages, limitations, and barriers to widespread adoption.

**Research question:**

What are surgeons’ perspectives of the exoscope compared to the OM?

**Materials and methods:**

A 29-question survey, designed by the authors, was used to compare the exoscope and the OM. The survey addressed key comparative aspects of both systems and included demographic information about participants and their institutions. Surgeons were recruited from internationally recognized organizations of expert spinal surgeons, with invitations sent via email. Responses were submitted anonymously, ensuring unbiased and authentic data collection.

**Results:**

Surgeons reported using the exoscope in a total of 1517 cases. Most surgeons found the exoscope comparable to or superior to the OM in many different parameters. 60.32 % of surgeons considered the exoscope to offer good learning opportunities, with most perceiving the learning curve as moderate (41.27 %) or mild (30.16 %). However, opinions on manoeuvrability and surgeon fatigue were divided—49.21 % found these aspects slightly better with the exoscope, a significant proportion felt they were worse. The primary drawback was its high cost, with additional concerns about ergonomics and resolution.

**Discussion and conclusion:**

From a surgeon's perspective, the exoscope presents several advantages over the OM. However, further improvements are needed, and it remains uncertain whether the exoscope can fully replace the OM in surgical practice.

## Highlights

• Most surgeons found the exoscope image quality comparable or superior.
• Most surgeons rated the exoscope learning curve as moderate or mild.
• 49.21 % of surgeons reported improved fatigue, while 36.51 % experienced worse.
• The primary limitation of exoscope use was the high cost (up to $1.5 million).
• 1.59 % of surgeons believed the exoscope could replace the operating microscope.

## 1 Introduction

The operating microscope (OM) has been a cornerstone of surgical visualization in spinal and neurosurgery since its introduction to neurosurgery in the 1950s (Kriss and Kriss, 1998; Milani et al., 2024). Over the decades, it has become the standard tool for enhancing surgical precision, offering magnification, illumination, and stereopsis—the perception of three-dimensional (3D) depth—through suspended binocular eyepieces (Ma and Fei, 2021; Vattipally et al., 2024). By providing a highly detailed view of the surgical field, the OM has significantly improved the accuracy of delicate procedures.

Despite its advantages, the OM is not without limitations. One of the key drawbacks is its restricted mobility, as the device is fixed in place and requires careful repositioning, which can be time-consuming during surgery (Vattipally et al., 2024; Iqbal et al., 2023; Ricciardi et al., 2019). Additionally, the OM is an expensive piece of equipment, which can limit accessibility, particularly in resource-constrained healthcare settings (Vattipally et al., 2024; Iqbal et al., 2023). Another limitation is the physical discomfort it may cause surgeons due to the need to maintain rigid postures for extended periods, which can contribute to musculoskeletal strain and fatigue (Ricciardi et al., 2019; Begagić et al., 2024; Fiani et al., 2021). Furthermore, surgeon interaction with the OM is somewhat limited, as only the primary operator directly views the surgical field through the eyepieces, potentially hindering collaborative efforts in the operating room (Vattipally et al., 2024).

**Fig. 1:**
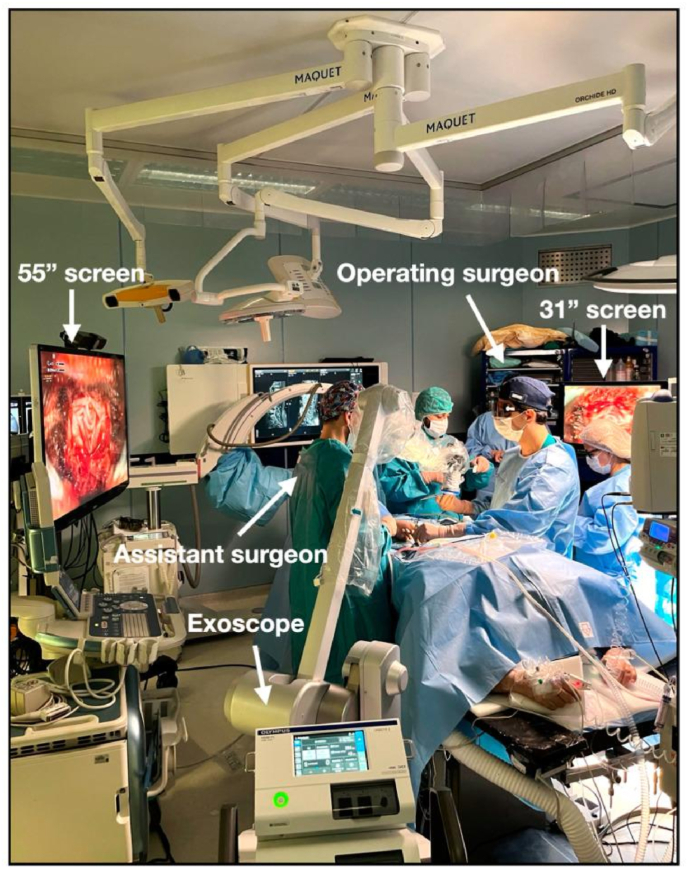
Image of Exoscope Set-Up for Operating Room. Adapted from Innocenti et al., 2024) under the Creative Commons Attribution-Non-Commercial License 4.0 (CCBY-NC) (12).

In response to these challenges, new surgical visualization tools have been developed over the years, leading to the introduction of the endoscope in the 1990s and, more recently, the exoscope (Schroeder and Sgouros, 2023; Kim et al., 2019). The exoscope was designed to address some of the inherent limitations of the OM while maintaining its benefits. Unlike the OM, which requires direct visualization through eyepieces, the exoscope employs a high-resolution telescopic camera, suspended above the surgical field, paired with an integrated light source (Vattipally et al., 2024; Ferreira et al., 2024). This camera captures and transmits a magnified, real-time image onto external monitors, allowing surgeons to operate while viewing a digital display rather than peering through a microscope (see Figure 1) (Vattipally et al., 2024; Begagić et al., 2024; Ferreira et al., 2024).

Earlier iterations of the exoscope were limited by their reliance on two-dimensional (2D) imaging, which could compromise depth perception and spatial awareness. However, newer models have been designed to better maintain stereopsis, using specialized 3D visualization technologies, such as glasses that enable depth perception when viewing the monitor screen (Vattipally et al., 2024). These advancements have positioned the exoscope as a promising alternative to the OM, potentially improving ergonomics, enhancing team collaboration by allowing multiple viewers to see the same surgical field, and offering greater flexibility in camera positioning (Vattipally et al., 2024; Begagić et al., 2024).

While numerous studies have explored the application of the exoscope in various surgical procedures, relatively few have focused exclusively on surgeons' perspectives regarding its advantages and limitations (Vattipally et al., 2024; Iqbal et al., 2023; Ricciardi et al., 2019; Begagić et al., 2024; Fiani et al., 2021). As the exoscope continues to gain traction in surgical settings, gathering feedback from surgeons who use this technology is essential for refining its design, optimizing its usability, and determining its potential to replace or complement the OM. This study aims to evaluate surgeons' opinions on how the exoscope compares to the OM, highlighting its benefits, drawbacks, and the challenges associated with its widespread adoption.

## 2 Materials and Methods

The survey methodology involved a comparative assessment of the exoscope and operating microscope (OM) using a 29-question survey designed by senior spine surgeons. The survey covered several key areas, including demographic information about the participants and their institutions, patterns of exoscope and OM use, comparative evaluations of both systems, and surgeons' opinions on their advantages and limitations (see Table 1).

Participants were openly recruited from internationally recognized organizations of expert spinal surgeons. Invitations were distributed via email, and respondents were given a four-week window to complete the survey. To ensure confidentiality and encourage candid feedback, all responses were submitted anonymously, minimizing potential bias and enhancing the authenticity of the data collected.

## 3 Results

### 3.1 Surgeon demographics

**Fig. 2:**
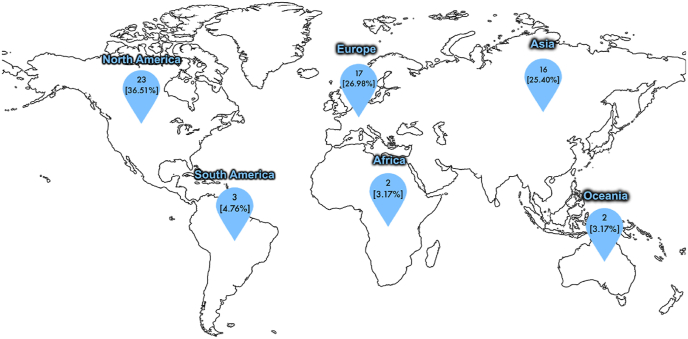
Map demonstrating where responding surgeons practice.

A total of 63 surgeons participated in the survey, comprising 25 neurosurgeons (39.68%) and 38 orthopaedic surgeons (60.32%). Surgeon respondents represented all six populated continents: 23 (36.51%) were from North America, 17 (26.98%) from Europe, 16 (25.40%) from Asia, 3 (4.76%) from South America, 2 (3.17%) from Africa, and 2 (3.17%) from Oceania (see Figure 2). Countries represented included the United States, Canada, the United Kingdom, Germany, Sweden, Switzerland, Japan, China, Brazil, and Australia. Some country names were omitted to preserve respondent anonymity.

The participating surgeons had a wide range of experience levels. Twenty-five (39.68%) had more than ten years of experience, 14 (22.22%) had six to ten years, 13 (20.63%) had one to five years, and 11 (17.46%) had less than one year of experience. Respondents were affiliated with a variety of institutions, including 26 (41.27%) from public institutions, 19 (30.16%) from mixed public-private institutions, and 18 (28.57%) from private institutions.

### 3.2 Exoscope and operating microscope use

As displayed in Table 2, the most used exoscopes were the Olympus ORBEYE™ and Zeiss KINEVO 900 (19 [30.16%] surgeons using each) and the most used OMs being the Leica M720 OH5 and Zeiss OPMI PENTERO 800 (19 [30.16%] surgeons using each).

**Table 1:** Survey questions.

Question	Response Options
How many years of practice as a Consultant/Attending Spine Surgeon best describes you?	a) less than one year b) 1–5 years c) 6–10 years d) more than 10 years
Which best describes your primary specialty of expertise?	a) Orthopaedics b) Neurosurgery
Where best describes your region of practice?	a) North America b) South America c) Europe d) Asia e) Africa f) Oceania
Please list below the country in which your institution or practice is located.	Open Ended
Which best defines your institution or practice in which the Exoscope is used?	a) Public b) Private c) Mixed
Please note below the exact Exoscope used in your institution or practice?	Open Ended
Please note below the exact OM used in your institution or practice?	Open Ended
How many cases would you estimate you have used the Exoscope for?	a) Less than 5 cases b) 6 to 10 cases c) 11 to 20 cases d) 21 to 30 cases e) More than 30 cases
If you know the exact number of cases you have used Exoscope for, please answer below. If you do not know or are unsure, please answer “NA”	Open Ended
Please describe below the type of procedures or cases (e.g. discectomy for lumbar disc herniation) you have used the Exoscope for. If known, please list case numbers beside procedure (e.g. lumbar disc herniation – 10).	Open Ended
Have you ever had to intraoperatively abandon use of the Exoscope and convert to OM/open procedure?	a) Yes b) No
If you answered “Yes” to above, please note below how many cases you had to abandon use of the Exoscope during a surgical procedure.	Open Ended
If you answered “Yes” to Question 9, please describe below the reasoning as to why you had to abandon use of the Exoscope during a surgical procedure.	Open Ended
How would you rate the learning curve associated with the Exoscope?	a) Minimal b) Mild c) Moderate d) High
How would you rate the training opportunities available for the Exoscope?	a) Poor b) Satisfactory c) Good d) Very Good
How does the image quality experienced with the Exoscope compare to that of the OM?	a) Significantly Better b) Slightly Better c) No Difference d) Slightly Worse e) Significantly Worse
How does the depth of accessibility in the surgical field experienced with the Exoscope compare to that of the OM?	a) Significantly Better b) Slightly Better c) No Difference d) Slightly Worse e) Significantly Worse
How does the manoeuvrability of the Exoscope compare to that of the OM?	a) Significantly Better b) Slightly Better c) No Difference d) Slightly Worse e) Significantly Worse
How does the magnification of the surgical field experienced with the Exoscope compare to that of the OM?	a) Significantly Better b) Slightly Better c) No Difference d) Slightly Worse e) Significantly Worse
How would you describe the length of comparative surgical procedures when employing the Exoscope compared to OM?	a) Significantly Improved b) Slightly Improved c) No Difference d) Slightly Longer e) Significantly Longer
If you answered significantly/slightly shorter/longer to the above question, what do you believe contributes to the discrepancy in operative time between the Exoscope and OM?	Open Ended
How would you describe surgeon fatigue of comparative surgical procedures when employing the Exoscope compared to OM?	a) Significantly Improved b) Slightly Improved c) No Difference d) Slightly Worse e) Significantly Worse
If you answered significantly/slightly improved/worse to the above question, what do you believe contributes to the discrepancy in surgeon fatigue between the	Open Ended
How would you describe surgeon eye strain of comparative surgical procedures when employing the Exoscope compared to OM?	a) Significantly Improved b) Slightly Improved c) No Difference d) Slightly Worse e) Significantly Worse
Does use of Exoscope aid/facilitate dual surgeon interaction in the surgical field compared to OM?	a) Yes b) No c) No Difference
Do you believe Exoscope will completely replace the use of OM in spinal procedures?	a) Agree b) Disagree c) Unsure
Please list below any further short-fallings, disadvantages, or limitations associated with the Exoscope.	Open Ended
Please list below any improvements you believe Exoscopes would benefit from as a practising spine surgeon.	Open Ended
Please list below any further comments you have in regards to the use of Exoscopes compared to OM in spinal procedures.	Open Ended

**Table 2:** Exoscopes and operating microscope used by respondents.

Exoscope Used	Number of Surgeons	OM Used	Number of Surgeons
Olympus ORBEYE™	19 (30.16%)	Leica M720 OH5	19 (30.16%)
Zeiss KINEVO 900	19 (30.16%)	Zeiss OPMI PENTERO 800	19 (30.16%)
Sony 4K3D	13 (20.63%)	Zeiss Tivato 700	13 (20.63%)
Leica M530 OHX	12 (19.05%)	Möller-Wedel	12 (19.05%)

A total of 1517 cases performed using the exoscope were reported by the surgeons. In 62 (4.09%) of those cases, the use of an exoscope had to be intraoperatively abandoned, and the surgeons converted to OM/open procedure, with the reason for abandonment for these cases being technical difficulty, including poor depth perception, limited manoeuvrability, and suboptimal visualization in deep surgical fields.

**Fig. 3:**
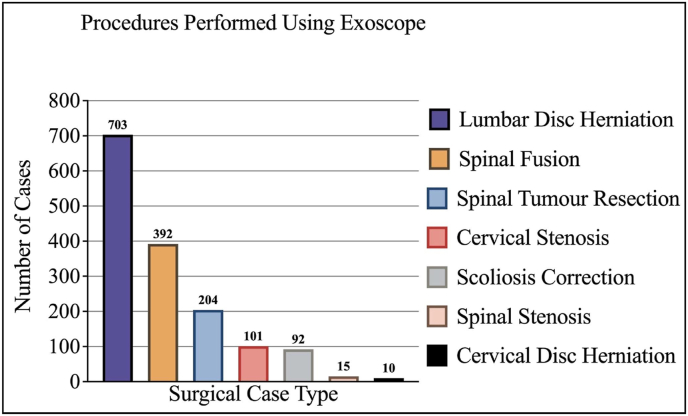
Procedures performed using exoscope.

Fig. 3 displays the different procedures the surgeons reported implementing the use of the exoscope. The vast majority of cases were lumbar disc herniation cases (703 [46.34%]), followed by spinal fusions (392 [25.84%]) and spinal tumour resections (204 [13.45%]). There were 101 (6.66%) cervical stenosis cases, 92 (6.06%) scoliosis correction cases, 15 (0.99%) spinal stenosis cases and 10 (0.66%) cervical disc herniation cases.

### 3.3 Exoscope Qualities compared to OM

**Fig. 4:**
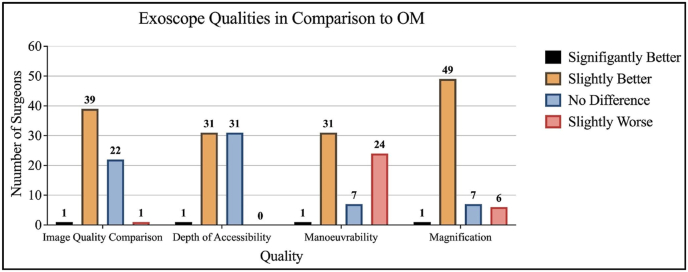
Exoscope qualities in comparison to OM

As shown in Fig. 4, most surgeons stated that the image quality comparison and the magnification of the exoscope were slightly better than the OM, with 39 (61.90%) of surgeons stating so for image quality and 49 (77.78%) of surgeons stating so for magnification. Regarding the depth of accessibility (referring to visualization in deep surgical fields or narrow corridors) of the exoscope, the surgeons' opinions were divided, with 31 (49.21%) surgeons stating that it was slightly better while 31 (49.21%) stated there was no difference. For manoeuvrability of the exoscope, most surgeons (31 [49.21%]) stated that it was slightly better, but a large number of surgeons (24 [38.10%]) also stated that it was slightly worse.

**Fig. 5:**
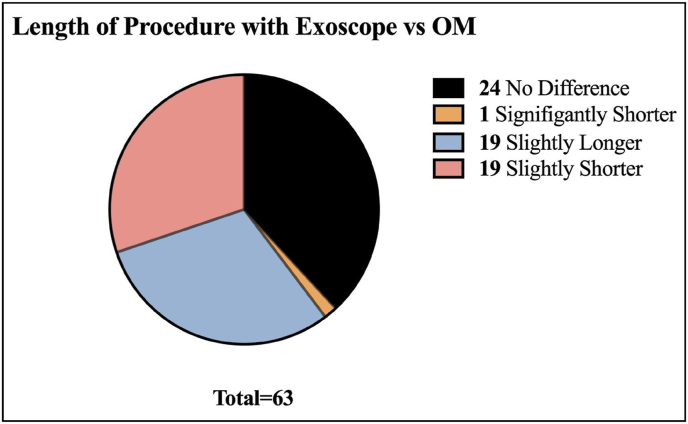
Graph Showing Exoscope vs OM Length of Procedure.

Regarding the length of procedure, most surgeons (24 [38.10%]) said there was no difference between the exoscope and OM, followed by 19 (30.16%) surgeons stating exoscope procedures were slightly longer and 19 (30.16%) stating the procedures were slightly shorter. 1 (1.59%) surgeon stated that the procedure was significantly shorter (see Figure 5). Of the 19 surgeons who stated the exoscope procedure times were slightly shorter, 18 stated that the time discrepancy was due to an improved workflow, while 1 stated it was due to the learning curve. The 1 surgeon who stated that the exoscope procedure time was significantly shorter stated it was due to improved workflow. Of the 19 surgeons who stated the exoscope procedure time was slightly longer, 2 stated it was due to a learning curve, and 17 stated it was due to technical issues.

**Fig. 6:**
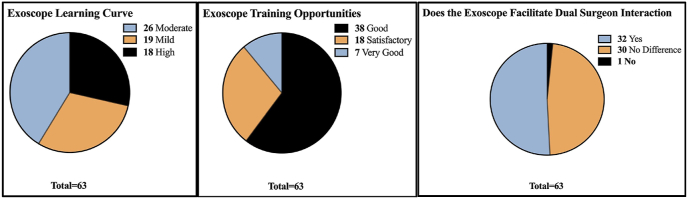
Graphs of exoscope learning, training and surgeon interaction qualities.

As shown in Table 3 and Fig. 6, most surgeons (26 [41.27%]) stated the learning curve for the exoscope was moderate followed by 19 (30.16%) finding it mild. Regarding training opportunities, most surgeons (38 [60.32%]) found the training opportunities with the exoscope to be good. When asked if the exoscope facilitated dual surgeon interaction in the surgical field. The opinion was split, with 32 (50.79%) surgeons responding yes and 30 (47.62%) responding that there was no difference from using an OM.

**Table 3:** Exoscope learning, training and surgeon interaction qualities.

Learning Curve	Number of Surgeons Responding	Training Opportunities	Number of Surgeons Responding	Dual Surgeon Interaction	Number of Surgeons Responding
High	18 (28.57 %)	Very Good	7 (11.11 %)	Yes	32 (50.79 %)
Moderate	26 (41.27 %)	Good	38 (60.32 %)	No	1 (1.59 %)
Mild	19 (30.16 %)	Satisfactory	18 (28.57 %)	No Difference	30 (47.62 %)

### 3.4 Surgeon fatigue and eye strain

**Fig. 7:**
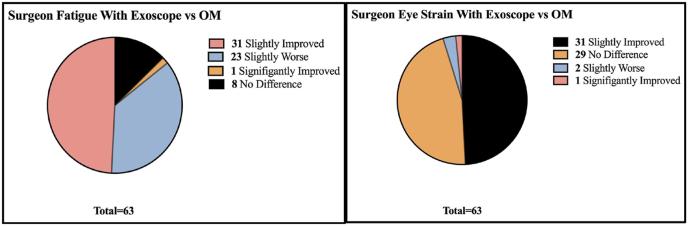
Graphs of exoscope surgeon fatigue and eye strain compared to OM

As displayed in Table 4 and Fig. 7 most surgeons (31 [49.21%]) found surgeon fatigue slightly improved; however many (23 [36.51%]) found the fatigue to be slightly worse. Those who stated the fatigue had improved stated it was due to better ergonomics, while those who found it worse stated it was due to poor ergonomics. For eye strain, most surgeons found no difference (29 [46.03%]) or that it was slightly improved (23 [36.51%]) when using the exoscope.

**Table 4:** Exoscope surgeon fatigue and eye strain compared to OM.

	Surgeon Fatigue	Surgeon Eye Strain
Significantly Improved	1 (1.59 %)	1 (1.59 %)
Slightly Improved	31 (49.21 %)	23 (36.51)
No Difference	8 (12.70 %)	29 (46.03 %)
Slightly Worse	23 (36.51)	2 (3.17 %)

### 3.5 Surgeon opinions

When asked if the exoscope would replace OM, 36 (57.14%) surgeons were unsure, 26 (41.27%) disagreed, and 1 (1.59%) agreed. Almost all surgeons (62 [98.41%] unanimously agreed that high cost was the main shortcoming of the exoscope, with the last 1 (1.59%) surgeon providing no answer. When asked what improvements were needed, 31 (49.21%) surgeons stated better ergonomics, 31 (49.21%) stated better resolution and 1 (1.59%) respondent provided no answer. Finally, only one surgeon commented that they highly recommend the exoscope.

## 4 Discussion

Exoscopes are being increasingly used in spinal and neurosurgery, as well as in many other surgical specialities (Vattipally et al., 2024; Garcia et al., 2023; Gaffuri et al., 2023). As the exoscope evolves as a new tool, surgeons' opinions can shape the development of newer models by highlighting advantages and limitations. In this study, the advantages of the exoscope noted by the respondents were the exoscope's superior magnification, depth of accessibility and image quality, as shown in Fig. 4. Other studies have shown similar results, with a systematic review by Iqbal et al. finding the exoscope has superior image quality and depth perception, and a systematic review by Ricciardi et al. found image quality and magnification to be at least equivalent to the OM (Iqbal et al., 2023; Ricciardi et al., 2019).

Another strength of the exoscope is its moderate learning curve and the training opportunities it provides as shown in Table 3. Our study reported that most surgeons found the learning curve to be mild or moderate, and many other studies have reported that the learning curve for the exoscope is shorter than that of the OM or the endoscope (Vattipally et al., 2024; Iqbal et al., 2023; Ferreira et al., 2024; Montemurro et al., 2021). One of the exoscopes main strengths in comparison to the OM is its potential for training junior surgeons. Due to its ability to provide high-resolution footage of the surgical field on monitors, the exoscope provides the opportunity for everyone in the operating room to see from the surgeon's perspective, which lends to training, education and teamwork (Vattipally et al., 2024; Ricciardi et al., 2019; Begagić et al., 2024; Fiani et al., 2021; Ferreira et al., 2024; Calloni et al., 2022). There is also the possibility of creating videos using the exoscope footage which would be able to supply detailed footage from the surgeon's perspective for training purposes without the requirement of being physically present in the operating room (Calloni et al., 2022). Videos made using the exoscope could provide further training for residents and documentation of rarer procedures (Calloni et al., 2022). Additionally, the exoscope facilitates more dual surgeon interaction than the OM as shown in Fig. 6.

Ergonomics are critical in surgery as operating in uncomfortable positions for prolonged periods can lead to musculoskeletal disorders (MSD) and pain (Schupper et al., 2023). Most studies regarding the exoscope report better ergonomics and less surgeon fatigue as one of the great benefits of the exoscope because surgeons are able to view the monitor at eye level and operate in a comfortable position (Vattipally et al., 2024; Iqbal et al., 2023; Ricciardi et al., 2019; Fiani et al., 2021; Ferreira et al., 2024; Montemurro et al., 2021; Schupper et al., 2023). A survey done by White et al. showed that high numbers of spine surgeons (70.4%) have experienced a MSD with 13.4% having to take time off work (White et al., 2024). MSDs can also influence early retirement or the restriction or modification of their practice (Epstein et al., 2018). While having a burden on the surgeon, MSDs also impact hospital costs and the lives of patients. For example, Hopkins et al. estimated the mean annual excess hospitalisation revenue of a neurosurgeon to be $5,120,533 USD and the annual hospital revenue in private practice groups to be $1,539,704 (Hopkins et al., 2024). Should surgeons retire early or require significant time off due to work-related MSDs or injury there would be a significant cost to the hospital. In this context the price of an exoscope ($250,000 to $1,500,000) may be warranted to improve surgeon ergonomics and reduce the prevalence of occupational-related MSDs (Fiani et al., 2021). However, while our study showed most surgeons (49.21%) did find the exoscope lessened surgeon fatigue, a significant number (36.51%), as shown in Table 4 and Fig. 7, found their fatigue to be worse due to poor ergonomics, with improvements necessary. Almost half of the respondents stated that one of the improvements needed is better ergonomics. This could potentially be solved by the reorganisation of the operating room to achieve the optimal position to view the monitors. As such, development of a standardized set-up that optimizes ergonomics may be beneficial.

Many studies report that eye strain is one of the limitations of the exoscope and also found that the 3D glasses used for 3D exoscopes may cause discomfort, headaches, and dizziness (Vattipally et al., 2024; Fiani et al., 2021; Ferreira et al., 2024; Montemurro et al., 2021). However, this study found that most surgeons viewed the eye strain with the exoscope to be equal to or improved compared to eye strain when using the OM as shown in Table 4 and Fig. 7. This difference could be due to differences in exoscope models. In Vatipally et al.‘s systematic review the majority of studies reporting side effects of headaches and nasal pain were using the Mitaka Kestrel View II model which was not one of the exoscopes used by surgeons in our study (Vattipally et al., 2024). The same exoscope model was used in the study mentioning the same side effects in Ferreira et al.‘s narrative review and was one of the studies included in Vatipally et al.‘s review (Ferreira et al., 2024; Yao et al., 2023).

Our study found that most surgeons thought the procedure length with the exoscope was equal to or slightly shorter than with the OM, which many other studies have also found (Begagić et al., 2024; Fiani et al., 2021; Ferreira et al., 2024; Montemurro et al., 2021). While the shorter procedure time may be an advantage of the exoscope many surgeons did respond that the operating time was slightly longer compared to the OM which is reflected in other studies finding exoscope operative lengths longer, potentially impacted by the learning curve (Milani et al., 2024; Iqbal et al., 2023). However, most surgeons reported that the increased procedure length was due to technical difficulties meaning further research into the issues surgeons are encountering using the exoscope may be valuable to improve the exoscope's technology.

As for limitations of the exoscope, almost all of the surgeons in our study thought that the main disadvantage of the exoscope was its high cost. Exoscopes are expensive equipment with costs ranging from $250,000 to $1,500,000. However, OMs also have considered capital cost, ranging from $250,000 to more than $750,000 (Ricciardi et al., 2019; Fiani et al., 2021). Additionally, Sarikonda et al. found that the intraoperative costs of the same procedure did not differ much between the exoscope and OM, with exoscope costs being $7951 +/− $3488 and OM costs being $6557 +/− $954 (Sarikonda et al., 2024). As OMs are naturally replaced, replacing the OM with an exoscope would not be more expensive than purchasing a new OM, depending on the model. Low-cost exoscopes also exist, with Encarnacion Ramirez et al. finding an exoscope they purchased for $150 an effective alternative to OM for Anterior Cervical Discectomy and Fusion (Encarnacion Ramirez et al., 2023). Therefore, while cost can be a limitation for the widespread use of advanced exoscopes, low-cost exoscopes may be an option for regions with less resources. Further research is needed concerning any capability compromise of such cost-attractive systems.

Beyond traditional high-resource surgical centres, one of the most impactful applications of exoscope technology may be its potential use as an independent alternative to the operating microscope in resource-limited or rural settings. Many of the survey respondents in this study represented a wide range of geographic and economic backgrounds, underscoring the need for adaptable, cost-effective visualization tools. In surgical environments where OM systems may be prohibitively expensive, difficult to maintain, or entirely unavailable, the exoscope could serve as a transformative solution; particularly if low-cost or portable models prove functionally adequate. This is further elucidated by the fact it has been described in the literature that low-cost exoscope adaptations that offer sufficient visualization for standard procedures at a fraction of the cost of traditional Oms (Encarnacion Ramirez et al., 2023). Furthermore, the exoscope's ability to project the surgical field onto screens may facilitate remote guidance or mentoring in settings with limited specialist access. While further validation is needed, especially regarding its standalone reliability in complex or deep-field procedures, the exoscope's potential to democratize surgical visualization and expand access in underserved areas represents a critical future direction for both research and health policy.

While many surgeons in our study found the manoeuvrability to be better with the exoscope (49.21%), many also found it slightly worse (38.10%). Other studies similarly had varied results on the manoeuvrability of the exoscope (Vattipally et al., 2024; Fiani et al., 2021). Fiani et al. suggest that the poor manoeuvrability is because manual repositioning of the exoscope mid-procedure is more difficult and time consuming than doing so with an OM as OMs have more advanced mechanisms (Fiani et al., 2021). However, manoeuvrability issues can be solved by using robotic exoscope systems for repositioning (Vattipally et al., 2024; Fiani et al., 2021). A final limitation of the exoscope that was found in our study was that almost half of the surgeons stated that the resolution of the exoscope could be improved. While being increasingly integrated in neurosurgery and spine surgery the exoscope has also been introduced into many other surgical fields including plastic and ear, nose and throat (E.N.T) surgery (Garcia et al., 2023; Gaffuri et al., 2023; Ferlito et al., 2022). Garcia et al.'s study in exoscope use in plastic surgery found the exoscope to be non-inferior to the OM in plastic microsurgery and found advantages and limitations similar to those in spine and neurosurgery (Garcia et al., 2023). Gaffuri et al. found similar results in the use of the exoscope in paediatric otorhinolaryngology with the exoscope proving to be safe and effective and having potential in the field (Gaffuri et al., 2023). Overall, the exoscope appears to be at least as effective as or with some advantages over the OM in the different surgical fields it has been implemented in. The division in opinion within both our survey respondents and what has been reported on in the literature may stem from differences in exoscope models, surgeon experience, differing utilities in different surgical specialties, or the presence or absence of robotic or motorized arm supports.

## 5 Study limitations and future directions

The major limitation of this study is respondent bias, as this study was an opinion-based survey. Surgeons may have been unable to recall the details of past cases they performed and may place more weight on their experience in their more recent cases. A solution to this would be to perform an ongoing prospective survey. Additionally, the majority of the respondents were from North America, Europe, and Asia, which may limit the study's generalisability. Moreover, as the exoscope is a relatively new piece of technology, how it compares in the long-term to the OM still needs to be determined.

Furthermore, while subgroup analysis based on the types of spinal procedures performed (e.g., cervical discectomy, thoracic instrumentation), as well as a correlational analysis (e.g., years of experience vs. exoscope preference) and logistic regression (for factors influencing satisfaction) would have provided greater clinical nuance, the structure and anonymization of our survey responses precluded this level of analysis. As procedural detail and other key values were not linked to individual surgeon responses, stratification by surgical indication and deploying a more robust statistical analysis was not feasible. Future prospective studies may benefit from designing survey instruments that allow for more detailed and structured analyses. Additionally, while this survey-based study did not directly measure complication rates, future research could include questions or analyses assessing surgeons' perceptions of whether exoscope use impacts intraoperative safety or complication risk. Understanding these perceptions may provide valuable insight into surgeon preferences and inform strategies for safe adoption of exoscope technology. Another limitation is that the survey did not differentiate between participants using 2D versus 3D exoscope systems. Since depth perception and eye strain may vary significantly between these modalities, future studies could stratify responses based on the imaging technology used to better assess user experience and preferences. Along with this, our study did not explore whether usage of the exoscope influences surgeons’ intraoperative clinical decisions. The enhanced visualization that surgeons can experience with the exoscope may affect the chosen surgical approach, extent of tissue dissection, intraoperative risk assessment, or even prompt a change in strategy during the procedure. Investigating these aspects in future studies is necessary.

Also, although surgeon fatigue and eye strain were assessed, these outcomes were based on subjective reporting rather than standardized metrics. Validated instruments such as the NASA Task Load Index (NASA-TLX) or the Visual Fatigue Scale were not utilized in this study. Incorporating such tools in future investigations would enhance the objectivity and comparability of these measures across different visualization platforms. Moreover, the usage of validated instruments like these in future studies would also allow for future investigation of the degree of motor and cognitive adaptation required when transitioning from the OM to the exoscope. Differences in visual orientation, hand-eye coordination, and depth estimation may present a unique set of challenges for surgeons adapting to exoscopic visualization—particularly those accustomed to direct binocular viewing through an OM. While our survey captured general impressions of ease of use, it did not formally evaluate the cognitive load or ergonomic strain associated with this transition. Future studies would benefit from structured assessments using validated tools such as the NASA Task Load Index (NASA-TLX) to quantify the mental and physical demands of this adjustment, as well as to identify user-specific factors that may predict smoother adaptation. Also, while the survey captured general perceptions of the learning curve associated with exoscope use, it did not collect data on the number of cases required for participants to feel proficient, nor did it assess how prior microscope experience may have influenced adaptation. This limits our ability to draw specific conclusions about the pace of skill acquisition. Future studies should consider incorporating these metrics to better characterize the exoscope's learning curve in relation to user background and case volume.

Another valuable future direction for this topic would be the implementation of a longitudinal follow-up survey to assess whether initial impressions of the exoscope translate into lasting preference or continued use over time. Tracking surgeons’ visualization tool preferences several months after initial exposure could provide important insights into long-term adoption patterns and the durability of early satisfaction.

## 6 Conclusion

Based on this study the exoscope is a promising tool in neurosurgery and spine surgery with it being found comparable to, or better than the OM in many categories. The advantages of the exoscope were its image quality, depth of accessibility, magnification, lesser degree of eye strain, training opportunities, better surgeon interaction and mild or moderate learning curve. Most surgeons found manoeuvrability and surgeon fatigue better with the exoscope, but many also found it worse. Similarly, most surgeons thought that the exoscope's length of procedure was equal to or better than the exoscope but a significant proportion found it to be slightly longer. Surgeons believed that the exoscope's main limitation was its high cost, and that improvement is needed in its ergonomics and resolution. From a surgeon's perspective, the exoscope offers several advantages over the OM. However, improvements to the exoscope are still needed and how the exoscope compares to the OM in the long-term is yet to be determined.

## Author contributions

All authors contributed equally to the conception and design of the study, data analysis and interpretation, and the drafting and revising of the manuscript. All authors have approved the final article for submission.

## Ethics statement

As this study involved an anonymous, non-sensitive survey with no collection of personal or health data, formal ethics approval was not required per institutional policy.

## Source of funding

This research did not receive any specific grant from funding agencies in the public, commercial, or not-for-profit sectors.

## Declaration of competing interest

The authors declare that they have no known competing financial interests or personal relationships that could have appeared to influence the work reported in this paper.
